# Patient Satisfaction With a Comprehensive Remote Care Program for Chronic Condition Management for Adults Under Medical Care: Observational Study

**DOI:** 10.2196/69586

**Published:** 2025-08-26

**Authors:** Michael Merrill, Susan M Zbikowski, Garrett J Jenkins

**Affiliations:** 1 Brook, Inc, Seattle, WA, United States; 2 inZights Consulting, LLC, 834 NE 95th Street, Seattle, WA, 98115, United States, 1 2063343723

**Keywords:** remote monitoring, patient satisfaction, chronic disease, self-management, telemedicine, digital health

## Abstract

**Background:**

More than half of adults in the United States have at least one chronic medical condition. Remote care strategies, including telemedicine, remote monitoring, and connected health devices, are increasingly used to support chronic condition management and adherence to provider-recommended care plans. Evaluating participant satisfaction and perceived usefulness is essential to understanding program value and potential for sustained engagement.

**Objective:**

This study aims to evaluate participant satisfaction, perceived usefulness, and self-reported health outcomes associated with a clinically deployed Remote Care program for chronic condition management that included nurse and coach follow-up, health monitoring devices, and digital education tools.

**Methods:**

A survey was emailed to 1411 active participants in the Brook Remote Care program between September 23 and December 8, 2023. The survey assessed satisfaction with program features, perceived usefulness, and self-reported health changes. Descriptive statistics were used to summarize the data, and chi-square tests were used to examine differences by age, gender, program duration, and health conditions.

**Results:**

A total of 360 participants completed the survey. Most participants rated the program as useful (320/359, 89%), and 66% (234/357) indicated they were likely to recommend it to others with similar health concerns. Between 68% (216/316) and 84% (277/332) rated specific program features as “good” to “excellent,” with nurse monitoring, health monitoring devices, and program cost receiving the most favorable ratings. Nearly half of participants reported improvements in physical health (155/314, 49%), reduced health-related stress (148/326, 45%), and increased knowledge of their condition (172/360, 48%). Additionally, approximately one-third reported improvements in lifestyle behaviors. Longer program duration was associated with higher satisfaction and greater self-reported health improvements.

**Conclusions:**

Participants reported high satisfaction with a remote care program designed to support chronic condition self-management. These findings support the value of integrated remote care models that combine device monitoring, personalized follow-up, and patient education to enhance patient experience and promote engagement in self-care.

## Introduction

Over half of adults in the United States have at least one chronic medical condition [1]. Most follow a medically prescribed treatment plan and adopt self-management strategies, including lifestyle changes and self-monitoring, to improve disease control and clinical outcomes [2]. Many chronic conditions also require frequent medical visits or hospitalization. Remote care, such as telehealth and monitoring with connected devices, offers an alternative to in-person visits, supporting condition management and reducing the risk of acute events and hospitalizations [3,4].

Remote care programs have been shown to reduce mortality, disease complications, hospitalizations, and associated health care costs across a range of medical conditions [5-7]. For example, one study involving patients with chronic obstructive pulmonary disease or congestive heart failure found a 68% and 35% reduction in emergency room visits and hospitalizations, respectively, over a 6-month period (comparing 3 months before and after the intervention) [6,7].

Patient satisfaction is a widely used quality metric, used both to evaluate care and meet regulatory reporting requirements. Understanding how patients experience remote care is essential for assessing its perceived value, ongoing engagement, and potential for broader adoption. A review by Kraai et al [8] reported high satisfaction with remote care, but most studies included in that review were pilot trials with varied designs and no evaluation of remote care offered in a routine clinical setting. A more recent study by Mayo Clinic researchers [9] found that patients receiving remote care for acute conditions, such as COVID-19, were highly satisfied with their care team and the usability of the technology. However, this study focused primarily on an acutely ill population, with 88% being treated for COVID-19, limiting generalizability to chronic care settings.

There remains a need to evaluate patient satisfaction with remote care programs designed specifically for community-dwelling individuals managing chronic diseases. This study aims to contribute to the literature by examining satisfaction and self-reported health changes among insured patients enrolled in a clinically delivered remote care program for chronic condition management.

## Methods

### Survey Administration

In September 2023, Brook Health emailed active participants (N=1411) in its digital Remote Care program, inviting them to complete a survey (administered via Google Forms) to assess their experience and satisfaction as part of ongoing quality assurance efforts. Two reminder emails were sent approximately 2 weeks apart, followed by a final reminder shortly before the survey closed—3 months after the initial invitation. To be eligible, individuals had to be actively enrolled in the Remote Care program and have an email address on file. Participation was voluntary. To encourage responses, participants were offered the opportunity to enter a drawing for 1 of 5 US $100 Amazon gift cards.

### Remote Care Program Description

The Remote Care program was developed and operated by Brook Health, a Seattle-based company that delivers digital health services to support clinic operations, provider-delivered care, and patient well-being. The program includes app-based educational resources and tools; wireless, cellular-enabled monitoring devices (scales, blood pressure cuffs, glucose monitors, and pulse oximeters) that do not require Bluetooth syncing; personalized one-on-one health coaching; and ongoing nurse monitoring to ensure care plan adherence and optimal condition management. Nurses monitored patient data and contacted clinics as needed to escalate care concerns. Patients were referred to the program by their providers and enrolled either onsite at the clinic or from home. After enrollment, patients received information about the program, downloaded the app, created an account, and were provided with digital monitoring devices. Participants received nurse monitoring and coaching calls throughout the program, with the highest frequency of contact occurring during the first month. Program costs were covered by insurance, with participants responsible for applicable copays.

### Survey Instrument

The survey assessed participants’ perceptions of program usefulness, satisfaction with specific features, and self-reported changes in health and well-being (refer to Textbox 1 for items). Brook developed a custom instrument informed by established digital health evaluation models, with an emphasis on face validity and alignment with program objectives. Acceptability was operationalized through participant experience, including user satisfaction ratings. Designed as a quality improvement tool for real-world implementation, the survey was intentionally brief and written in accessible language to accommodate a diverse patient population. Formal psychometric testing was not conducted. In addition to items assessing specific program components, the survey included a general self-rated health item from the 20-item Short Form Survey and a global health change item modeled after the Global Rating of Change scale, a widely used patient-reported outcome measure [10,11].

Textbox 1. **Participant survey questions for the Brook Remote Care Program.**

**Program ratings**

How would you rate the following aspects of the Brook Remote Care program? Very poor, poor, average/acceptable, good, or excellent.

- Ease of enrolling
- Program content
- Nurse interactions
- Coaching
- Devices provided
- Cost of the program
- Program duration
- Results/outcomes

How would you rate the following aspects of Nurse Monitoring in the Remote Care program? Very poor, poor, average/acceptable, good, or excellent.

- Quality of interactions with the nurse
- How often the nurse contacted you
- Amount of time spent on calls with the nurse
- Recommendations made by the nurse

On a scale from 1 to 10, how likely are you to recommend this program to others with similar health concerns? (1=Not all to 10=Extremely likely)

Rate how useful Brook Remote Care has been in helping you to manage your health/health condition? Extremely, very, somewhat, a little, or not at all.

**Health ratings**

In general, would you say your health is: excellent, very good, good, fair, or poor?

Would you say your health has improved, not changed at all, or worsened since joining Brook Remote Care?

Since joining Brook Remote Care, how do you feel when it comes to stressing about your health status?

While participating in Brook Remote Care, did you...? Check all that apply.

⬜ Achieve better control of your health condition

⬜ Lose weight

⬜ Increase physical activity

⬜ Eat healthier

⬜ Play a more active role in your health

⬜ Reduce medication use

⬜ Reduce stress/feel less anxious

⬜ Sleep better/more

⬜ Become happier

⬜ Become more knowledgeable about your condition and management

⬜ Spend less time on medical appointment and tests

⬜ None of the above/other

How has your use of medications to manage your condition (for which you were referred to Remote Care) changed since joining the program? Check all that apply.

⬜ Not on any medications for my condition (never took or stopped taking before starting Brook)

⬜ No change in my medication use

⬜ I started taking medication(s) for my condition

⬜ Changed the type of medication

⬜ Decreased amount, strength or frequency

⬜ Increased amount, strength, or frequency

**Descriptives**

How long have you been participating in Brook Remote Care? Less than 1 month, 1-3 months, 4-6 months, 7-9 months, 10-12 months, or more than 12 months.

For which medical condition are you being supported by Brook Remote Care? Congestive heart failure, diabetes, hypertension, weight loss, or other.

Age in years: 18-24, 25-34, 35-44, 45-54, 55-64, 65-74, or 75+.

Your gender: female, male, nonbinary, or transgender.

### Ethical Considerations

Participation was voluntary. The following information was included with the survey. “Your responses to this survey are confidential. Your answers will not be shared with your provider or anyone else. Brook Health will only use your responses in aggregate with responses from others who complete this survey. By answering this survey, we presume your approval to use your responses for the following purposes...1) improve existing services, 2) inform the development of new services, 3) create marketing materials, or 4) generate research reports.” To encourage responses, participants were offered the opportunity to enter a drawing for 1 of 5 US $100 Amazon gift cards. This study was reviewed by the University at Buffalo institutional review board and deemed exempt from full review (IRB ID STUDY00008350).

### Sample

A power calculation determined that 302 completed surveys were needed to achieve a statistically representative sample with 95% confidence and a 5% margin of error. A total of 441 surveys were received. During data cleaning, 81 duplicate responses were identified and removed; specifically, only the first complete response from each participant (based on the timestamp of submission) was retained. This resulted in 360 unique surveys included in the final analysis, yielding a response rate of 26%.

### Descriptive Analysis

Descriptive statistics were calculated for all demographic variables and survey items. Chi-square analyses were conducted to examine group differences in ratings of program features, nurse monitoring, overall program usefulness, likelihood of recommending the program, and self-reported health status and health changes. Comparisons were made across gender (male vs female), age (<65 vs 65+ years), selected health conditions (hypertension, diabetes, obesity, and congestive heart failure), and program duration (<6 months, 7‐12 months, and 12+ months). In addition, multivariate logistic regression models were conducted to assess whether observed associations remained statistically significant after adjusting for age, gender, and program duration, accounting for potential confounding factors.

### Item Scoring

The Net Promoter Score (NPS), a widely used measure of overall program experience, was derived from the question: “On a scale from 1 to 10, how likely are you to recommend this program to others with similar health concerns?” (1=not at all likely; 10=extremely likely). Respondents were categorized as Promoters (scores of 9‐10), Passives (scores of 7‐8), or Detractors (scores of 6 or below). The NPS was calculated as the percentage of Promoters minus the percentage of Detractors. Positive NPS values indicate that more participants would recommend the program than not, while negative scores suggest the opposite [12].

## Results

### Sample Characteristics

Most participants identified as female (223/359, 62%), were aged 65 years or older (251/359, 70%), and had a single chronic condition (220/359, 61%), most commonly hypertension (266/359, 74%). Just more than half (204/3, 57%) had been enrolled in the program for less than 6 months (Table 1).

**Table 1.** Survey respondent demographics.

Demographics	Values (N=359), n (%)
Gender (N=359)	
Female	223 (62)
Male	136 (38)
Age (years; n=359)	
<35	4 (1)
35‐44	18 (2)
45‐54	25 (7)
55‐64	61 (17)
65‐74	178 (50)
75+	73 (20)
Health condition^a^	
Single	220 (61)
Multiple	140 (39)
Hypertension	266 (74)
Obesity	127 (35)
Diabetes	98 (27)
Congestive heart failure	27 (8)
Other	20 (6)
Duration in the program (months)	
<6	204 (57)
7‐12	99 (28)
12+	56 (16)

a Multiresponse item. Participants could report multiple health conditions.

### Self-Reported Health and Health-Related Changes

Most respondents rated their health as “good” or better (263/358, 74%). Nearly half reported improvements in their physical health (155/314, 49%) and a reduction in health-related stress (148/326, 45%) since enrolling in the program. Reported behavior and condition management improvements included increased knowledge of their condition (172/360, 48%), more active involvement in their care (143/360, 40%), healthier eating (120/360, 33%), improved condition control (116/360, 32%), increased physical activity (112/360, 31%), and reduced stress (96/360, 27%; Table 2).

**Table 2.** Survey respondents’ self-reported health and health-related changes.

Health-related items	Values, n (%)
Health status (n=358)	
Poor	14 (4)
Fair	81 (23)
Good	186 (52)
Very good	67 (19)
Excellent	10 (3)
Health change since enrolling (n=314)	
Improved	155 (49)
Same	153 (49)
Worsened	6 (2)
Health-related stress (n=326)	
Less	148 (45)
Same	156 (48)
More	22 (7)
Reported improvements (n=360)	
Knowledge	172 (48)
More active role in health	143 (40)
Eat healthier	120 (33)
Better condition control	116 (32)
Lose weight	108 (30)
Increase physical activity	112 (31)
Reduce stress	96 (27)
Sleep better	52 (14)
Happier	39 (11)
Less time on medical appointments/tests	28 (8)
Medication change (n=356)	
Decrease	42 (12)
Increase	40 (11)
Started/changed medication	65 (18)
Not on any/no change	235 (66)

### Program Ratings

**Figure 1.**
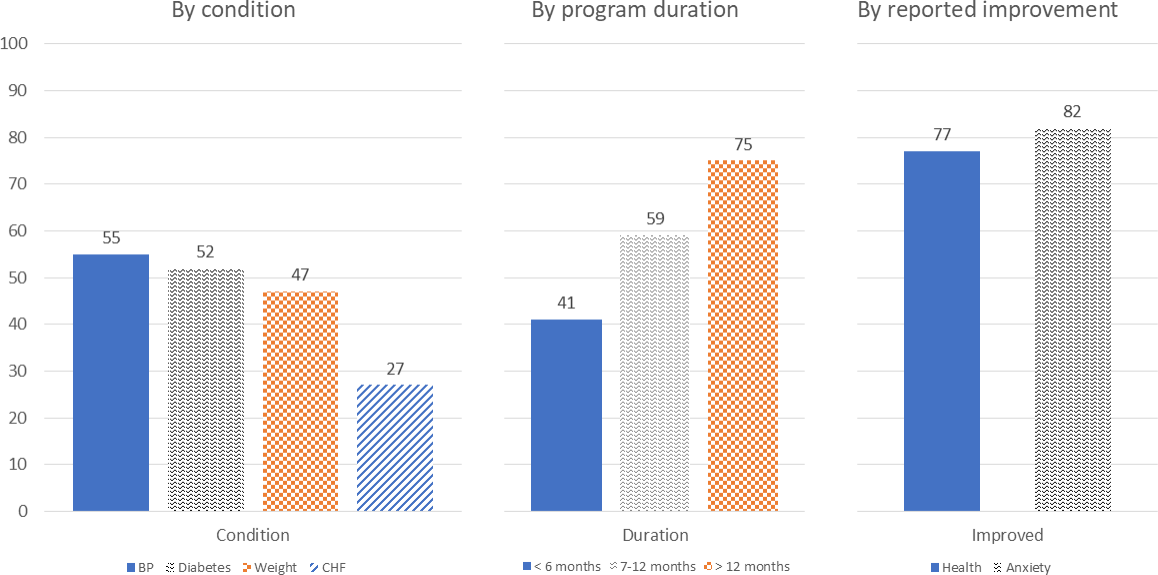
Net Promoter Scores by condition, program duration, and reported health improvements. BP: hypertension; CHF: congestive heart failure.

The majority of participants (320/359, 89%) rated the Remote Care program as “somewhat” to “extremely” useful. Based on responses to the NPS item, 66% (234/357) were classified as Promoters, 20% (72/357) as Passives, and 14% (51/357) as Detractors, yielding an overall NPS of 52, categorized as “excellent.” NPS was higher among those with longer enrollment, self-reported health improvements, and individuals managing diabetes or hypertension (Figure 1).

### Program Feature Ratings

All Remote Care program features received favorable ratings, with most respondents rating components as “good” to “excellent.” Nurse monitoring, remote monitoring devices, and program cost received the highest “excellent” ratings (>50%). Coaching, educational content, and outcomes were rated more variably, with a broader distribution across “average,” “good,” and “excellent” ratings. Fewer than 7% of respondents rated any feature as “poor” or “very poor” (Table 3).

**Table 3.** Program participant responses related to aspects of the Brook Remote Care program and nurse monitoring.

Questions (number of respondents)	Answers					
	Excellent, n (%)	Good, n (%)	Average/ acceptable, n (%)	Poor, n (%)	Very poor, n (%)	Good to excellent, %
Aspects of Brook Remote Care Program						
Ease of enrolling (n=332)	155 (47)	122 (37)	46 (14)	4 (1)	5 (2)	84
Content (n=322)	115 (36)	138 (43)	57 (18)	7 (2)	5 (2)	79
Nurse interactions (n=331)	176 (53)	99 (30)	45 (14)	7 (2)	4 (1)	83
Coaching (n=316)	118 (37)	98 (31)	78 (25)	15 (5)	7 (2)	68
Devices (n=330)	175 (53)	98 (30)	49 (15)	3 (1)	5 (1)	83
Program cost (n=317)	204 (64)	62 (20)	40 (13)	6 (2)	5 (2)	84
Program duration (n=307)	120 (39)	111 (36)	69 (22)	3 (1)	4 (1)	71
Outcomes (n=312)	113 (36)	112 (36)	72 (23)	7 (2)	8 (3)	72
Aspect of nurse monitoring						
Quality of interaction (n=333)	183 (55)	83 (25)	51 (15)	10 (3)	6 (2)	80
Frequency of contact (n=331)	152 (46)	93 (28)	70 (21)	13 (4)	3 (1)	74
Amount of time spent during calls (n=329)	149 (45)	96 (29)	67 (20)	11 (3)	6 (2)	74
Recommendations made by nurse (n=326)	148 (45)	91 (28)	65 (20)	17 (5)	5 (2)	73

### Comparative Analyses by Participant Characteristics

Chi-square analyses indicated that self-reported physical health improvements were significantly associated with age (*χ*²_1_=10.56; *P*=.001) and program duration (*χ*²_2_=8.72; *P*=.01). Reductions in health-related stress differed by gender (*χ*²_1_=7.18; *P*=.007) and program duration (*χ*²_2_=5.99; *P*=.05), with trends also observed for age (*χ*²_1_=3.62; *P*=.06). Participants enrolled longer reported higher satisfaction across several program components, including ease of enrollment, content, duration, and perceived outcomes (all *P*<.05). Nurse monitoring ratings were also significantly higher among those with longer program participation (*χ*²_4_=10.49; *P*=.02). No significant differences in program or nurse ratings were found by health condition. A nonsignificant trend suggested men rated nurse recommendations more positively than women (*χ*²_4_=5.54; *P*=.06), and younger adults rated overall program usefulness slightly higher (*χ*²_2_=4.87; *P*=.09). Multivariate logistic regression models including age, gender, and program duration were conducted to adjust for potential confounding factors. Multivariate findings were consistent with univariate results, with no substantial changes in the significance or direction of observed associations.

## Discussion

### Principal Findings

This study found high participant satisfaction with a clinically implemented Remote Care program designed to support chronic condition management through digital monitoring, nurse outreach, and personalized coaching. Most participants reported the program was useful in helping manage their health, and satisfaction was highest among those with longer program engagement. These findings add to a growing body of literature supporting the acceptability and perceived value of remote care solutions for patients with chronic disease.

Participants reported a variety of benefits beyond satisfaction, including improvements in physical and emotional health, condition-related knowledge, and self-management behaviors. These findings are consistent with prior studies showing that remote care can support healthier lifestyles and improved condition control [6,13]. While the observational design limits causal conclusions, the high percentage of participants reporting improvements, particularly in knowledge and engagement, suggests the program may support behavior change in a real-world clinical setting.

Program duration emerged as a consistent factor associated with greater perceived benefit. Participants who were enrolled longer reported more health improvements, higher satisfaction ratings, and stronger endorsement of program features, including nurse support and educational content. While the direction of this relationship is unclear, whether satisfaction encourages continued engagement or vice versa, this aligns with prior research showing satisfaction and engagement are mutually reinforcing and essential to program effectiveness [14].

Few differences in satisfaction emerged across demographic groups. While age and gender were associated with some trends in health perception and nurse rating preferences, program usefulness and satisfaction ratings did not vary significantly by gender, age, or specific health condition. Notably, men, who often engage less with health coaching, rated nurse recommendations particularly favorably, suggesting an opportunity to better tailor outreach or messaging to male participants. Although the association did not reach significance, this pattern may indicate that nurse-led outreach is especially effective for engaging men in self-management—a group often underrepresented in such programs. This warrants further exploration.

Younger adults also appeared to perceive the program as slightly more useful, a pattern that contrasts with previous findings where older adults more often rate remote tools favorably [9]. This may reflect generational differences in expectations for digital health and could inform strategies to optimize engagement across age cohorts. While these trends fell short of statistical significance, they may still carry clinical relevance, particularly given their consistency with prior research and potential value in refining remote care models. In real-world settings with modest sample sizes, patterns like these may not reach conventional significance thresholds yet still highlight meaningful opportunities to personalize care and improve patient experience.

Nurse monitoring was highly rated and is likely a core driver of overall satisfaction. One-on-one nurse interactions may have contributed directly to the reported improvements in condition knowledge, stress reduction, and behavior change. Prior studies support the conclusion that structured remote care with personalized nurse support can enhance self-management and improve outcomes for patients with chronic illness [5,13].

The NPS, an indicator of participant willingness to recommend the program to others, was in the “excellent” range. Importantly, this finding came from a sample with a full range of user experiences, including detractors and passives, not just those who were highly satisfied. This suggests that the program delivered a consistently positive experience across a diverse patient population.

### Why This Is Meaningful

Remote care has grown in popularity, especially following the COVID-19 pandemic, as health care systems seek scalable approaches to support chronic disease management [4]. Evidence suggests that remote care and telemonitoring can improve patient outcomes and reduce health care use, including emergency department visits and hospitalizations [5-7]. As these programs become more widely adopted, understanding the patient experience is critical to sustaining engagement and maximizing impact.

This study contributes to the growing body of literature showing high satisfaction with remote care programs [8,9] and extends it by focusing on a clinically integrated program for patients with chronic, not acute, conditions. The consistently high satisfaction and NPSs across diverse subgroups indicate that user-centered remote care models are feasible, scalable, and well-suited for long-term condition management.

### Limitations

This study has several limitations. First, outcomes are based on subjective, self-reported data collected from a single group of participants, without a comparison group. This limits our ability to determine whether the observed outcomes were attributable to the program itself or reflect the natural course of condition management.

Second, the survey had a response rate of 26%. It is possible that the experiences and satisfaction levels of survey respondents differ from those who did not respond. However, the distribution of NPSs suggests a range of perspectives was captured. Specifically, 66% (234/357) of respondents were classified as “Promoters,” 20% (72/357) as “Passives,” and 14% (51/357) as “Detractors,” indicating the survey captured both positive and critical feedback about participants’ likelihood to recommend the program.

Third, the high satisfaction ratings related to program cost are likely influenced by the fact that the program was covered by insurance for nearly all participants. This may have bolstered overall satisfaction scores. It is unclear whether participants would have rated the program as highly if they were required to pay out of pocket. Prior research has shown that cost is a frequent barrier to program use [15].

Fourth, while some demographic and health-related differences were observed between survey responders and nonresponders, these were modest and not suggestive of systematic response bias; nonetheless, the possibility of unmeasured differences affecting the generalizability of findings cannot be ruled out.

Finally, the demographic data available for analysis were limited to age, gender, condition, and program duration. We did not have access to important clinical characteristics such as duration of diagnosis or disease severity. Future studies should consider collecting more detailed clinical data to better characterize the participant population and contextualize outcomes.

Because the survey was developed for quality improvement purposes and not psychometrically validated, the reliability and comparability of the findings may be limited. However, the use of face-valid items aligned with program goals supports their relevance for capturing participant experience in real-world care settings.

### Conclusions

This study demonstrates that patients managing chronic conditions within a clinically delivered Remote Care program reported high levels of satisfaction, perceived usefulness, and self-reported health improvements. Longer duration in the program was consistently associated with greater perceived benefit, highlighting the value of sustained engagement.

The findings suggest that remote care programs incorporating regular nurse outreach, connected health devices, and tailored educational content can support patients in managing their health outside traditional clinical settings. As health care systems continue to adopt digital and hybrid care models, understanding patient experience will remain essential to driving meaningful outcomes. Future research should incorporate longitudinal or comparative designs to evaluate the program’s clinical effectiveness and long-term impact on behavior change and health outcomes.

## Abbreviations

NPS: Net Promoter Score
SF-20: 20-item Short Form Survey
